# Reducing consumer level food waste through message framing: A systematic review and social marketing interventions

**DOI:** 10.1177/0734242X251369629

**Published:** 2025-09-19

**Authors:** Melissa Zulu, Asphat Muposhi

**Affiliations:** Marketing Division, School of Business Sciences, University of the Witwatersrand, Johannesburg, South Africa

**Keywords:** Food waste reduction, consumer-level food waste, message frames, construal levels, social marketing, systematic literature review

## Abstract

Food waste is characterised as a complex, wicked and multi-faceted problem with adverse environmental, social and economic implications. One way of addressing this challenge is to enhance the effectiveness of messages employed to promote food waste reduction behaviour. This study reviews literature on message frames employed to address the problem of food waste at consumption settings such as households, restaurants and hotels. A systematic literature review of quantitative, qualitative and mixed method studies focusing on consumer level food waste reduction was conducted. With the aid of the Preferred Reporting Items for Systematic Reviews and Meta-Analyses (PRISMA) framework, 33 studies published between 2015 and 2024 were reviewed. Thematic content analysis was used to conduct data analysis. The use of multiple food waste reduction message frames was identified as a growing trend, although there is lack of consensus on the best integration approach. The effectiveness of food reduction message frames was found to be influenced by differences in consumers’ construal levels, message design, behaviour inducements and consumption context. Messages that blame consumers for wasting food were found to have a backfiring effect that triggers resistance to engage in food waste reduction initiatives. The results of this study underscore the importance of developing an integrated social marketing communication intervention that incorporates multiple message frames. The prevalence of different construal levels associated with food waste affirms the role of segmentation, targeting and positioning in the development of food waste reduction intervention programmes.

## Introduction

Food waste is one of the contemporary grand challenges confronting humanity (Abos et al., 2024; Zhang et al., 2020). Food waste refers to any food that is appropriate for human consumption but is wasted or discarded (Dai and Gong, 2024). Food waste is characterised as a complex, wicked and multi-faceted problem with adverse economic and social implications (United Nations Environment Programme [UNEP], 2021). The global economic cost associated with food waste per year is estimated to be US$1 trillion (UNEP, 2021). Food wasted compromises food security because it represents a missed opportunity to sustain livelihoods of underprivileged communities, especially in developing economies (FAO, 2021). As a response to the grim consequences of food waste, the United Nations, through Sustainable Development Goal 12.3, committed to halve per capita global food waste by 2030 (UNEP, 2021). Although food waste occurs at all stages of the supply chain, it is regarded as more pervasive at the consumption stage (Khalil et al., 2021; Zhang et al., 2020). Food consumption settings such as households, hotels and restaurants are identified as major contributors of consumer-level food waste (UNEP, 2021). Globally, it is estimated that consumers contribute to almost 88% to the amount of food wasted (UNEP, 2024). It is for this reason that more concerted efforts are being directed towards reducing consumer-level food waste in consumption settings such as households, restaurants and hotels (Antonschmidt and Lund-Durlacher, 2021; Begho and Zhao, 2023).

Changing consumer behaviour especially in consumption settings such as households, restaurants and hotels where food is considered as a pleasurable experience is identified as the long-term strategy for reducing food waste (Begho and Zhao, 2023; Dolnicar et al., 2020; Khalil et al., 2022). Social marketing is regarded as an effective behavioural change strategy with the ability to redirect consumer attitudes and behaviours to support food waste reduction initiatives (Abos et al., 2024; Ong et al., 2023). Food waste is regarded as a social marketing issue because it has adverse implications on society and consumer well-being (Pearson and Perera, 2018). In the context of food waste, social marketing is operationalised through message framing which involves the development and dissemination of messages that promote food waste reduction behaviour (Abos et al., 2024). The major advantage of social marketing over alternative interventions such as food rationing is that it promotes voluntary self-regulated behaviour which lessens the administrative burden and consumer resentment (Ong et al., 2023; Pearson and Perera, 2018). Moreover, Stöckli et al. (2018) noted that the use of involuntary strategies such as restricted portion sizes often generate social criticism which trigger consumer resistance. Due to its ability to promote voluntary behaviour, social marketing is generally recommended as a food waste reduction strategy (Abos et al., 2024; Ong et al., 2023).

Although the role of message framing has been widely examined as a strategy to reduce consumer-level food waste (e.g., Abos et al., 2024; Khalil et al., 2022; Xu and Jeong, 2019; Zhang et al., 2020), mixed results were reported. For example, inconsistent findings were reported regarding the effectiveness of message frames, with some studies reporting that positive messages have a higher impact (Bretter et al., 2023; Dai and Gong, 2024; Grazzini et al., 2018), whereas others argue that negative messages frames are more effective (Huang et al., 2021; Jiang et al., 2024a; Zheng et al., 2023). Although notable effort has been expended in framing messages that can be used to address food waste, Khalil et al. (2022) as well as Abos et al. (2024) argued that the question of how to effectively convey these messages remains unanswered. Concurring with this view, Dai and Gong (2024) also observed that the persuasive effect of messages employed to reduce food waste remains limited and often fail to grab the attention of consumers. In order to harmonise insights from previous studies, this study employs a systematic literature review (SLR) with a specific focus on message framing as a food waste reduction strategy.

Although several SLRs were conducted to harness best practices to reduce consumer level food waste (e.g., Dhir et al., 2020; Kasavan et al., 2022; Vizzoto et al., 2020), SLRs that specifically focus on message framing as a food waste reduction strategy are limited. Notable SLRs with a focus on message framing were conducted by Florence et al. (2022) and Heiges et al. (2022). Florence et al. (2022) adopted a broader perspective with a focus on several types of sustainable behaviours, and this makes it a challenge to delineate context specific interventions. A review by Heiges et al. (2022) included consumption settings such as schools and university canteens where food is regarded as a necessity (Dolnicar et al., 2020). According to Dolnicar et al. (2020), findings from such consumption settings may not accurately reflect the effectiveness of food waste reduction messages. As food is considered as a necessity in schools and university canteens, the findings may not be generalised to consumption settings such as household and hotels which are often characterised with the perception of food abundance and pursuit of hedonic benefits (Dhir et al., 2020; Vizzoto et al., 2020). This study addresses this research gap by conducting an SLR of message frames employed to address food waste at household level and in the hospitality industry.

An SLR focusing on message framing is necessary because, as noted by Khalil et al. (2021) and Ong et al. (2023) consumers’ reluctance to engage in behavioural change, as shown by the attitude–behaviour gap, remains a major impediment towards food waste reduction. The attitude–behaviour gap is more prevalent in the hospitality sector and at household level where the desire for hedonic behaviours such as pleasure and perception of abundance are common (Grazzini et al., 2018; Huang et al., 2021). Given the complexities associated with addressing the challenge of food waste (Abos et al., 2024; Zhang et al., 2020) coupled with the prevalence of a wide array of message frames, Zhang et al. (2020) noted that formulating effective behavioural change messages remains a challenge for social marketers and policymakers. This challenge accentuates the need to review and synthesise extant literature in order to glean valuable insights required to develop effective food waste reduction messages. This study addresses the following research question: Is message framing effective as a strategy of reducing food waste in the hospitality industry? To address this research question, the following specific objectives were formulated:

1. To examine the types of food waste reduction message frames employed in literature
2. To explore the consumer construal levels associated with the problem of food waste
3. To identify the most appropriate food waste reduction message frame(s) recommended in extant literature

This study contributes to food waste research by reviewing literature on food waste and message framing with the objective of capacitating social marketers to develop consistent, coordinated and comprehensive messages to address the multi-faceted problem of food waste. Based on insights gained from the reviewed literature, this study proposes an integrated food waste reduction model which include the key enabling and constraining factors. The next section discusses the link between food waste and social marketing as well as the underpinning theories.

## Literature review

### Food waste reduction and social marketing

Food waste is defined as either unavoidable or avoidable loss that occurs in the food supply chain (Pearson and Perera, 2018). Unavoidable food waste refers to any food which is considered improper for human consumption based on food safety standards (Grazzini et al., 2018). Conversely, avoidable food waste, which is of interest to this study, refers to any food which is disposed of while still suitable for human consumption (Leverenz et al., 2019). Consumers’ perception of food waste depends on economic considerations, safety, security, degree of environmental concern and the associated construal level (Leverenz et al., 2019). Food waste reduction is regarded as a social marketing issue because it contributes to social equity by availing more food to the needy and improve quality of life by promoting environmental sustainability (Abos et al., 2024; Pearson and Perera, 2018).

Due to the characterisation of food waste as a social problem, policymakers often rely on social marketing messages to educate consumers to understand the importance of minimising food waste (Ong et al., 2023; Pearson and Perera, 2018). Social marketers employ several message frames such as gain, loss, informative, environmental and normative to promote food waste reduction (Abos et al., 2024; Grazzini et al., 2018; Ong et al., 2023). Although this effort is commendable, Abos et al. (2024) and Ong et al. (2023) underscored the need to continuously evaluate the effectiveness of such messages. Social marketing seeks to enhance behavioural change and as such the successes of message framing in this study is measured by the decrease in food waste.

### Underpinning theories

Food waste reduction message frames are posited to have a symbiotic and interactional relationship with consumer construal levels (Grazzini et al., 2018; Zhang et al., 2020). Informed by this view, this study is underpinned by the prospect theory (Tversky and Kahneman, 1981) and the construal level theory (Trope and Liberman, 2012). The prospect theory posits that individuals assess the appropriateness of behavioural actions based on anticipated outcomes which are framed on a gain to loss continuum (Tversky and Kahneman, 1981). In the context of food waste, a gain-frame strategy reinforces the benefits associated with food waste reduction (Grazzini et al., 2018). Food security and decrease in carbon emissions are the key gains associated with food waste reduction. Conversely, a loss-frame strategy emphasises the negative outcomes associated with increased levels of food waste such as hunger (Zhang et al., 2020). In order to elicit favourable behavioural change outcomes, it follows that social marketers need to understand the effectiveness of using either positively or negatively framed food waste reduction messages.

Construal level theory postulates that perceived psychological distance influences an individual’s attitudes and performance of recommended behaviours (Trope and Liberman, 2012). One of the challenges associated with framing effective messages to address environmental problems such as food waste is to address the perception of temporal and social distance (Xu and Jeong, 2024; Zhao et al., 2023). The construal level theory is therefore applied in this study to argue that consumers interpret food waste reduction messages based on their attribution of psychological distance. Drawing from the construal level theory (Trope and Liberman, 2012), dimensions such as social distance, time, space and probability of occurrence are used to identify construal levels incorporated in food waste reduction message frames in this study. Consumers’ interaction with the adverse effects of food waste such as hunger, poverty and social inequalities play a key role in influencing response behaviour (Xu and Jeong, 2024; Zhang et al., 2020). The natural environment is regarded as a public good and free rider behaviour is a common challenge in environmental sustainability studies (Muposhi and Mugwati, 2024). Moreover, some consumers perceive that the goal of attaining environmental sustainability is not urgent (Muposhi and Mugwati, 2024). Therefore, we argue that such perceptions if not addressed have the potential to increase the psychological distance associated with food waste reduction. The next section outlines the research methodology.

## Materials and methods

An SLR of food waste reduction message frames was done to address the research objectives. An SLR was employed because it is considered to be more rigorous and bias free as scientific procedures are followed to select relevant studies (Saunders et al., 2019). The use of SLR as a research method is consistent with previous studies on food waste research (Dhir et al., 2020 and Florence et al., 2022). The SLR was conducted following the Preferred Reporting Items for Systematic Reviews and Meta-Analyses (PRISMA) guidelines (Moher et al., 2009; Page et al., 2021). The use of the PRISMA guidelines enhanced credibility and rigour as all studies were systematically included or excluded in line with the searching criteria, timeframes and research objectives (Page et al., 2021).

### Search strategy

Published peer-reviewed studies on food waste message framing were identified through searches of EBSCO, ProQuest, Scopus, Science Direct and Web of Science databases. These databases were selected for SLR because they are the most commonly used repositories for multi-disciplinary studies such as food waste. The search period ranged from September 2015 up to September 2024. The year 2015 was selected because it coincides with the adoption of Sustainable Development Goals 2 and 12 which identified food waste as one of the global challenges (United Nations, 2021). A relatively long search period 2015–2024 was deliberately selected with the objective of capturing more studies on food waste message framing. The search terms along with connectors ‘OR’ and ‘AND’ were as follows: (‘food waste reduction’ OR ‘food waste mitigation’ OR ‘food waste prevention’) AND (‘food waste message framing’ OR ‘food waste message frames’) AND (‘consumer behaviour’ OR ‘hospitality industry food waste’). These search terms were searched in the titles, abstracts and full texts of selected studies.

### Screening procedures

This study only reviewed empirical studies that examined the impact of message frames on food waste reduction strategy in consumption settings such as households, hotels and restaurants. Studies that were conducted in schools and university canteens were excluded because consumption in such settings is principally driven by utilitarian needs (Dolnicar et al., 2020), and it may be difficult to have an objective assessment of the effectiveness of food waste reduction messages. English studies employing qualitative, quantitative or mixed methods were considered. Moreover, only studies with food waste reduction as a behavioural outcome were considered. Consistent with the approach followed by Kim et al. (2019), to enhance validity, book chapters and conference papers were excluded from the analysis.

**Figure 1.**
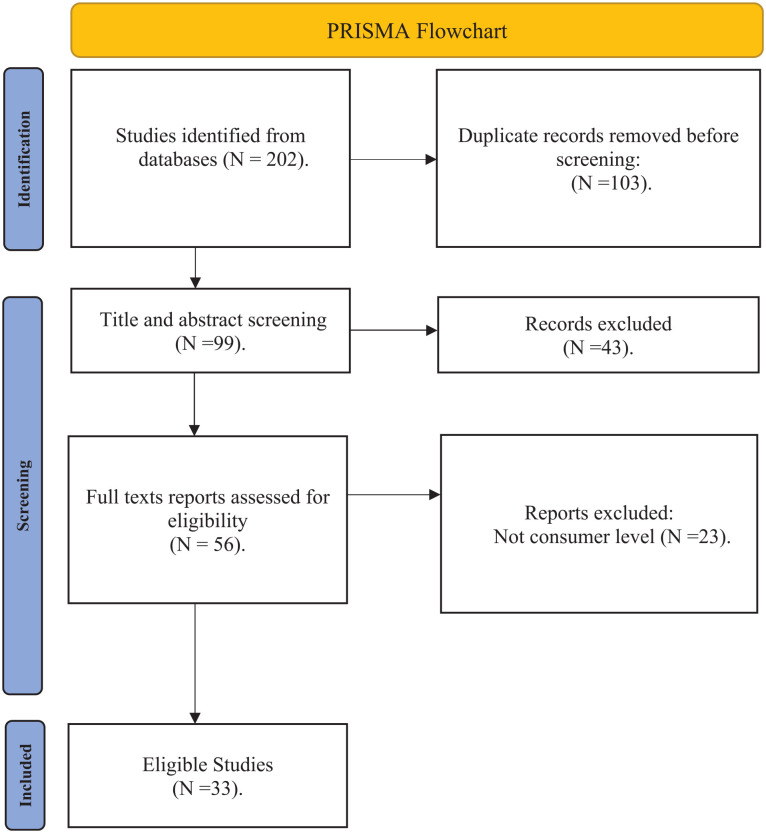
PRISMA flowchart. Source: Authors’ compilation. PRISMA: Preferred Reporting Items for Systematic Reviews and Meta-Analyses.

The initial search yielded 201 studies on food waste reduction and message frames. Prior to the screening of the identified studies, a total of 103 duplicates were removed. Three reviewers with expertise in food waste and message framing independently assessed the selected studies. Any discrepancies that emerged were resolved by analysing the full article. Firstly, the titles and abstracts of 98 studies were screened by checking the coverage of key search words such as message framing and food waste reduction. A total of 43 studies were excluded because of inadequate coverage of the search terms or phrases. After examining the full texts of the 55 remaining studies, a further 23 studies were dropped because their focus was either on organisational or supply chain food waste reduction behaviour. Ultimately, 33 studies were eligible for analysis. Figure 1 shows the PRISMA flowchart which was used to select eligible studies.

### Data extraction

The extraction of data from the selected 33 studies considered the types of message frames, methodology, use of theory and behavioural outcomes. Table 1 shows the profile of selected articles.

**Table 1.** Profile of selected studies.

Journal	Authors	No. of articles
*Journal of Retailing and Consumer Services*	Khalil et al. (2021)	4
	Zheng et al. (2023)	
	Dai and Gong (2024)	
	Jiang et al. (2024b)	
*Journal of Cleaner Production*	Antonschmidt and Lund-Durlacher (2021)	4
	Bretter et al. (2023)	
	Van Herpen et al. (2023)	
	Jiang et al. (2024a)	
*Resources, Conservation and Recycling*	Prelez et al. (2023)	3
	Wharton et al. (2021)	
	Stöckli et al. (2018)	
*International Journal of Contemporary Hospitality Management*	Xu and Jeong (2019)	2
	Olavarria-Key et al. (2021)	
*Journal of Social Marketing*	Ong et al. (2023)	2
	Abos et al. (2024)	
*Journal of Business Research*	Khalil et al. (2022)	2
	Birau and Faure (2018)	
*Journal of Sustainable Tourism*	Grazzini et al. (2018)	2
	Huang et al. (2021)	
*Journal of Quality Assurance in Hospitality & Tourism*	Chen and Jai (2018)	2
	Chen and DeSalvo (2022)	
*Current Issues in Tourism*	Chang (2022)	1
*Tourism Management*	Dolnicar et al. (2020)	1
*British Food Journal*	Cozzio et al. (2021)	1
*Environmental Science and Policy*	Nisa et al. (2022)	1
*Journal of Hospitality and Tourism Management*	Xu and Jeong (2024)	1
*Journal of Travel & Tourism Marketing*	Zhao et al. (2023)	1
*Food Frontiers*	Begho and Zhao (2023)	1
*Journal of Foodservice Business Research*	Schäufele-Elbers et al. (2024)	1
*International Journal of Hospitality Management*	Lei et al. (2024)	1
*Food Quality Preference*	Gimenez et al. (2023)	1
*Waste Management*	Matzembacher et al. (2020)	1
*Environment Behaviour*	Van der Werf et al. (2019)	1
Total articles analysed		33

Source: Authors’ compilation.

### Data analysis

Consistent with previous SLR studies (e.g., Dhir et al., 2020; Florence et al., 2022; Kaur et al., 2021), data were analysed using content analysis. To minimise bias, three reviewers independently analysed the data following a three-staged procedure. Firstly, open coding whereby reviewers sifted through the data was performed with the objective of identifying patterns, concepts and sub-themes (Braun and Clarke, 2016). The second stage involved axial coding which is an inductive and deductive process of grouping and categorisation of categories that emerged from open coding with the objective of developing themes (Creswell, 2009). To avoid omission of key themes, as recommended by Braun and Clarke (2016), an iterative process was followed whereby emergent themes were compared with research objectives. The last staged involved theme integration and refinement where the focus was on ensuring that the emergent themes are consistent with the data (Creswell, 2021). To ensure that the emergent themes are a true reflection of the data, three independent reviewers cross-checked the themes and consensus was established. Ultimately, the following themes emerged from the data analysis: *Types of message frames, message frame design, construal levels, role of context* and *behavioural outcomes.*

## Results and discussion

This section presents and discusses the results of SLR with a focus on the characteristics of the selected studies, types of message frames, message design, construal levels, role context and food waste reduction behavioural outcomes.

### Characteristics of selected studies

As shown in Table 2, the majority of studies were conducted in developed economies. The USA accounted for the majority of the studies (*n* = 12), followed by China (*n* = 6) and United Kingdom (*n* = 3). There were no empirical studies on message framing and food waste reduction that were identified in low-to-middle income economies. This is so despite the fact that the effects of food waste are more adverse in low-to-middle income economies due to fragile food security systems (Food Waste Index, 2024). As shown in Table 2, field experiments, controlled experiments and online experiments were the most dominant research methods. Cozzio et al. (2021) cautioned that the use of simulated environments may fail to capture the nuances associated with food waste reduction in natural consumption settings. The majority of the studies applied the prospect theory and construal level theory. Other theories that were employed include the theory of planned behaviour, framing theory, nudge theory, hope theory, alphabet theory and elaboration likelihood model. Table 2 shows the characteristics of selected studies.

**Table 2.** Characteristics of selected studies.

Authors	Study context	Research method	Theory
Abos et al. (2024)	Australia	Online focus group and quantitative survey	Elaboration likelihood model
Gimenez et al. (2023)	Australia	Quasi-experiment	Social practice theory; theory of planned behaviour
Antonschmidt and Lund-Durlacher (2021)	Austria	Quasi-experiment	Attitude-behaviour-context theory
Matzembacher et al. (2020)	Brazil	Semi-structured interviews	–
Van der Werf et al. (2019)	Canada	Randomised experiment	Theory of planned behaviour
Dai and Gong (2024)	China	Experiment	Limited capacity model
Zhao et al. (2023)	China	Scenario-based experiments	Framing theory and construal level theory
Zheng et al. (2023)	China	Experiment	Nudge theory
Lei et al. (2024)	China	Scenario-based experiments	Regulatory focus theory
Jiang et al. (2024a)	China	Scenario-based experiment	Prospect theory, expected utility theory
Jiang et al. (2024b)	China	Field experiment	Prospect theory, expected utility theory and level of explanation theory
Birau and Faure (2018)	Europe	Scenario-based experiments	Attribution theory
Cozzio et al. (2021)	Italy	Field experiment	–
Schäufele-Elbers et al. (2024)	Italy	Quasi-experiment	Alphabet theory
Ong et al. (2023)	Malaysia	Field experiment	Nudge theory
Van Herpen et al. (2023)	Netherlands	Quasi-experiment	Motivation-opportunity-ability model
Prelez et al. (2023)	Spain	Online experiment	Theory of planned behaviour
Bretter et al. (2023)	UK	Field experiment	–
Begho and Zhao (2023)	UK	Online survey	Prospect theory
Grazzini et al. (2018)	UK	Field experiment	Prospect theory and construal level theory
Chen and DeSalvo (2022)	USA	Field experiment	Prospect theory and framing theory
Chen and Jai (2018)	USA	Online experiment	Prospect theory
Nisa et al. (2022)	USA	Online experiments	–
Huang et al. (2021)	USA	Online experiments	Prospect theory, construal level theory
Khalil et al. (2022)	USA	Field experiment and questionnaire	Prospect theory and hope theory
Khalil et al. (2021)	USA	Experiment	Prospect theory
Olavarria-Key et al. (2021)	USA	Focus groups and quasi experiment	–
Xu and Jeong (2024)	USA	Experiment	Construal level theory
Xu and Jeong (2019)	USA	Experiment	Construal level theory
Zhang et al. (2020)	USA	Online experiment	Regulatory focus theory and construal level theory
Line et al. (2016)	USA	Online survey and scenario-based experiments	Construal level theory and information processing theory
Wharton et al. (2021)	USA	Quasi-experiment	Theory of planned behaviour
Dolnicar et al. (2020)	Slovenia	Quasi-experimental field	–
Chang (2022)	Taiwan	Field experiment	–

Source: Authors’ compilation.

### Types of message frames

The results of SLR revealed that there are several message frames that are used to structure food reduction messages. As shown in Table 3, most studies used the gain versus loss message frame, followed by the environmental versus societal frame and the positive versus negative information frame. As indicated in Table 3, use of single frame and paired message frames emerged as the most common approaches used to frame food waste reduction messages. The use of a single message frame was criticised for failing to consider an integrative approach which is regarded as effective to address the multifaceted problem of food waste (Begho and Zhao, 2023; Dai and Gong, 2024; Nisa et al., 2022). The results of SLR also revealed the prevalence of mixed views on the most effective way of pairing food waste reduction message frames (Chen and Jai, 2018; Schäufele-Elbers et al., 2024; Zheng et al., 2023). The most common message pairing combinations that emerged from literature reviewed include gain versus loss, environmental versus societal frame and positive versus negative information frame.

**Table 3.** Types of message frames.

Message frame	Studies	Number of studies that cited the message frame(s)
Gain versus loss	Begho and Zhao (2023); Dai and Gong (2024); Grazzini et al. (2018); Huang et al. (2021); Jiang et al. (2024a); Khalil et al. (2022); Lei et al. (2024); Prelez et al. (2023); Van der Werf et al. (2019); Zhao et al. (2023)	10
Environmental and societal frame	Bretter et al. (2023); Chang (2022); Chen and DeSalvo (2022); Chen and Jai (2018); Huang et al. (2021); Nisa et al. (2022); Pearson et al. (2017)	7
Positive versus negative information frame	Chen and DeSalvo (2022); Ong et al. (2023); Schäufele-Elbers et al. (2024)	3
Experiential message frame	Abos et al. (2024); Cozzio et al. (2021); Olavarria-Key et al. (2021)	3
Emotional frame	Birau and Faure (2018); Jiang et al. (2024b); Khalil et al. (2022)	3
Informative message frame	Gimenez et al. (2023); Stöckli et al. (2018)	2
Informational versus emotional versus financial (rewards vs penalties).	Jiang et al. (2024b); Matzembacher et al. (2020)	2
Environmental and financial frame	Bretter et al. (2023); Van der Werf et al. (2019)	2
Moral and financial	Chang (2022); Van Herpen et al. (2023)	2
Environmental frame	Dolnicar et al. (2020); Line et al. (2016)	2
Environmental messages, graphic and written messages, context manipulation	Antonschmidt and Lund-Durlacher (2021); Wharton et al. (2021)	2
Positive versus negative information versus normative message frame	Zheng et al. (2023)	1
Prevention versus promotion frame	Zhang et al. (2020)	1
Emotional versus rational	Zhao et al. (2023)	1
Attribute versus benefit frame	Xu and Jeong (2024)	1
Gain versus loss and informative frame	Khalil et al. (2021)	1

Source: Authors’ compilation.

The gain versus loss frame emphasises the cost savings or the financial burden associated with food waste (Bretter et al., 2023). The environmental versus societal frame emphasises the environmental and social benefits of saving food such as reduction of waste directed to landfills and food security (Chen and DeSalvo, 2022; Huang et al., 2021). The positive versus negative information frame focuses on disseminating educational, persuasive and instructive food waste reduction messages (e.g., Jiang et al., 2024a; Ong et al., 2023). Through the concept of moral suasion (Stöckli et al., 2018; Zheng et al., 2023), the normative frame seeks to promote self-regulated food waste reduction behaviour by harnessing the power of personal and social norms. Experiential message frame focuses on the use of action and participation oriented message frames (Abos et al., 2024; Olavarria-Key et al., 2021). Lastly, Jiang et al. (2024b), Khalil et al. (2021) and Zheng et al. (2023) employed an integrative approach that combines gain, loss and informative message frames. Integrated message frames were found to be more effective than single message frames (Begho and Zhao, 2023; Khalil et al., 2021). Table 3 summarises the message frames used to reduce food waste at household level and in the hospitality industry.

### Message frames and construal levels

The effectiveness of food reduction messages was also found to be influenced by the differences in consumers’ construal levels (Grazzini et al., 2018; Huang et al., 2021; Zhang et al., 2020). Despite the key role played by construal levels in influencing food waste reduction, only a few studies incorporated them. Construal level theory dimensions (Trope and Liberman, 2012) such as social distance, time, space and probability of occurrence were used to identify construal levels incorporated in food waste reduction message frames. Table 4 summarises the construal levels incorporated in food waste reduction messages at household level and in the hospitality industry.

**Table 4.** Message frames and construal levels.

Type of construal level	Studies	Impact
Self versus others versus environment	Chen and Jai (2018); Huang et al. (2021); Stöckli et al. (2018)	Gain versus loss messages that employ self versus others versus environment as points of references were found to be more effective in promoting food waste reduction as they evoke perceptions of corporate social responsibility
Self- versus others	Zhang et al. (2020); Zhao et al. (2023)	Food waste reduction messages that focus on individual benefits were found to be more effective as compared to those emphasising on collective societal benefits.
Proximal social distance	Zhao et al. (2023)	Gain-framed messages with a far social distance were found to reduce food waste than gain-framed messages with proximal social distance. Loss-framed message with a proximal social distance were found to effective in reducing food waste than loss-framed messages with far social distance.
Past versus future	Xu and Jeong (2024)	Messages that emphasise future social benefits were found to be more effective in reducing food waste than messages focusing on past achievements.
Close actors versus distant actors	Birau and Faure (2018)	Guilt framed messages that blame social actors that are close to consumers targeted with behavioural changes messages were found to result in ambivalent attitudes towards food waste reduction.
Concrete versus abstract	Grazzini et al. (2018)	Loss framed messages paired with a concrete construal level were found to be more effective in empowering consumers on how to reduce food waste.
Local versus distant	Line et al. (2016)	Food waste reduction messages that emphasise local benefits were found to be more effective as compared to those that focus on issues that are perceived as distant such as climate change.

Source: Authors’ compilation.

### Message frame designs

The SLR also revealed that the effectiveness of food waste reduction messages depends on how they are designed (Grazzini et al., 2018; Olavarria-Key et al., 2021). Table 5 summarises the message design factors that emerged from literature reviewed.

**Table 5.** Message frame designs.

Message design format	Studies	Impact
Experiential and anthropomorphic cues	Cozzio et al. (2021)	Participation based experiential cues were found to be more effective in capacitating consumers with practical skills to address food waste.
Written versus verbal	Olavarria-Key et al. (2021)	Consumers with low levels of mindfulness were found to respond unfavourably to food waste reduction messages delivered verbally as compared to written messages.
Visual cues and infographics	Antonschmidt and Lund-Durlacher (2021); Cozzio et al. (2021); Wharton et al. (2021)	Visual cues were found to evoke more positive imaginations and sensations related to food waste reduction behaviour.
Graphic versus written	Abos et al. (2024)	Food waste reduction messages in graphical format were found to create more consumer attention than text-based messages.
Cuteness cues	Lei et al. (2024)	Playful emoji incorporated into menus was found to positively influence food waste reduction behaviour.

Source: Authors’ compilation.

### Message frame context

The results of SRL also revealed that consumers’ receptivity to food waste reduction messages is significantly influenced by contextual factors (Antonschmidt and Lund-Durlacher, 2021; Dolnicar et al., 2020). The effectiveness of contextual cues was however found to be influenced by the way they are positioned in consumption settings (Antonschmidt and Lund-Durlacher, 2021; Dolnicar et al., 2020; Xu and Jeong, 2019). For example, a study by Xu and Jeong (2019) found that proximity to a social context facing food insecurity where individuals can visualise the adverse impact of food waste motivates the performance of food waste reduction behaviours. The placement of food waste reduction messages such as visual reminders at consumer contact points such as ‘entrances’ to the hotel or restaurant, ‘buffet’ and ‘guest table’ was found to be effective in communicating food waste reduction messages (Abos et al., 2024; Antonschmidt and Lund-Durlacher, 2021). Although the role of context played a significant role in consumption settings such as hotels and restaurants, its role in a household setting was reported to be minimal (Chen and DeSalvo, 2022; Nisa et al., 2022).

### Mediators and moderators of food waste reduction message frames

Mixed results emerged on the effectiveness of message frames and construal levels (e.g., Dai and Gong, 2024; Olavarria-Key et al., 2021; Zhao et al., 2023). To address this challenge, a strand of literature reviewed (e.g., Chen and Jai, 2018; Dai and Gong, 2024; Huang et al., 2021; Xu and Jeong, 2024) emphasise the need to consider the role of mediating and moderating factors in influencing consumers’ reception of food waste reduction messages. Table 6 summarises factors that mediate food waste reduction messages.

**Table 6.** Mediators factors of food waste reduction messages.

Mediator	Author(s)	Impact
Corporate social responsibility	Huang et al. (2021)	Food waste reduction messages are more effective when framed as part of a hotel’ corporate social responsibility initiatives.
Perceived trustworthiness	Xu and Jeong (2024)	Food waste reduction messages from sources perceived as credible were found to grab the attention of consumers as compared to those from untrusted sources.
Perceived intrinsic motives	Chen and Jai (2018); Olavarria-Key et al. (2021); Zhang et al. (2020)	Intrinsic factors such as attitude, mindfulness and perceived ability influence the reception of food waste reduction messages.
Emotional states	Dai and Gong (2024); Khalil et al. (2022)	Consumers driven by the emotional states of hope and anxiety show greater willingness to reduce food waste.
Evaluative self-reactions	Dai and Gong (2024)	Consumers are more likely to engage in food waste reduction when they favourably evaluate their ability to reduce food waste.

Source: Authors’ compilation.

Moreover, the literature reviewed also revealed the prevalence of factors that constrain or weaken the effectiveness of food waste reduction messages. Table 7 provides moderating factors of food waste reduction messages.

**Table 7.** Moderators of food waste reduction messages.

Moderator	Author(s)	Impact
Construal levels	Grazzini et al. (2018)	Construal levels were found to moderate the effectiveness of food waste reduction messages.
Green involvement	Zhao et al. (2023)	Consumers with a higher level of environment concern were found to respond more favourably to food waste reduction messages as compared to those with lower levels of environment concern.
Social responsible consumption	Chen and Jai (2018)	Consumers with higher level of social responsible consumption respond more favourably to food waste reduction messages.
Habits	Prelez et al. (2023)	Food waste reduction habits positively influence consumers’ responses to food waste reduction messages.
Perceived task difficulty	Birau and Faure (2018)	Perceived task difficulty was reported to influence consumers’ perception of food reduction messages.
Financial inducements	Chang (2022); Schäufele-Elbers et al. (2024)	Financial nudges were found to have a positive influence on consumers’ reception of food waste reduction messages.
Social norms	Zheng et al. (2023)	Social norms were reported to moderate the relationship between food waste messages and food waste reduction behaviour.
Context	Zheng et al. (2023)	The perception of food waste reduction messages was found to be influenced by the consumption context.

Source: Authors’ compilation.

### Message frames and food waste reduction behaviour

The SLR revealed mixed results on the effect of message frames on food waste reduction behaviour. One stream of literature reported that loss framed messages with an emphasis on financial loss are more effective in promoting food waste reduction behaviour (Begho and Zhao, 2023; Chen and DeSalvo, 2022; Khalil et al., 2021). Loss framed messages were reported to be more effective when supported with accurate quantitative data showing the magnitude of costs associated with food waste (Khalil et al., 2021). Another corpus of literature reviewed framed food reduction behaviour as a social issue. Normative message frames were found to be more effective in reducing food waste when social norms are internalised as personal norms and foster self-regulated food reduction behaviours (Stöckli et al., 2018; Zheng et al., 2023).

Other studies (e.g., Antonschmidt and Lund-Durlacher, 2021; Dolnicar et al., 2020; Xu and Jeong, 2019) argue that food waste reduction messages should be complemented with contextual cues in order to be effective. To address the multifaceted problem of food waste, another stream of literature (e.g., Jiang et al., 2024b; Khalil et al., 2021; Zheng et al., 2023) propose the use of multiple message frames in order to convey a consistent and unified food waste reduction message in a cost-effective manner. Table 8 summarises key findings on the impact of message frames on food waste reduction behaviour from selected studies.

**Table 8.** Impact of message frames on food waste reduction behaviour.

Reference	Message frame	Key findings
Line et al. (2016)	Environmental frame	Consumers respond favourably to food waste reduction messages that are consistent with their environmental values.
Birau and Faure (2018)	Emotional frame	Guilt framed messages trigger consumers’ negative attitudes towards food waste reduction.
Grazzini et al. (2018)	Gain versus loss	A concrete food waste reduction message combined with a loss-framed message was found to enhance consumers’ perceptions of self-efficacy to reduce food waste.
Stöckli et al. (2018)	Informative message frame	Exposure to informative food waste reduction prompts resulted in more diners to request to take their leftovers home.
Xu and Jeong (2024)	Attribute-based framed messages	Benefit-based messages were found to be more effective in reducing food waste as compared to attribute-based framed messages.
Dolnicar et al. (2020)	Environmental frame	Written environmental messages were found to be more effective in reducing food waste.
Cozzio et al. (2021)	Experiential message frame	Experiential appeals encompassing emotional, visual and participatory cues were more effective in reducing food waste as compared to normative and informative messages.
Khalil et al. (2021)	Gain versus loss and informative frame	A loss framed message accompanied with the precise numerical quantification of food wasted was found to be more effective in reducing food waste.
Huang et al. (2021)	Gain versus loss	A gain versus loss framed message accompanied by self versus others versus environment construal levels was found to reduce food waste as it evokes the perception that the restaurant is socially responsible.
Cozzio et al. (2021)	Experiential message frame	Participatory cues that reinforce altruistic values persuaded tourists to reduce food waste.
Chen and DeSalvo (2022)	Positive versus negative information frame	Positive food waste messages were found to trigger favourable attitudes towards food waste reduction as they convey positive outcomes associated with food waste reduction.
Khalil et al. (2022)	Gain versus loss	Gain (vs loss) framed messages elicited hope that the challenge of food waste can be addressed and this increased food waste reduction intention.
Begho and Zhao (2023).	Gain versus loss	A loss framed message with a focus on financial implications and a gain framed message with an emphasis on environmental benefits were found to be effective in reducing food waste at household level.
Prelez et al. (2023).	Gain versus loss	Co-benefits message framing positively influenced food reduction behaviour of environmentally concerned consumers.
Zheng et al. (2023).	Positive versus negative information versus normative message frame.	Negative framed messages were found to be more effective when paired with injunctive norms. Positive framed messages were more effective when paired with descriptive norms. A negative-descriptive norm framed message was found to have a backfiring effect that increases food waste behaviour.
Zhao et al. (2023).	Emotional versus rational	Loss-framed messages were found to be more effective in reducing food waste as compared to gain-framed messages. Food waste reduction messages with emotional appeals were reported to be more effective in reducing food waste than messages employing rational appeals.
Abos et al. (2024).	Experiential message frame	The use of visual cues was found to be more effective in reducing food waste.
Schäufele-Elbers et al. (2024).	Positive versus negative information frame	The use of information nudges resulted in food waste reduction behaviour even after the implementation phase.

Source: Authors’ compilation.

### Theoretical contributions

This study enriches literature on consumer-level food waste reduction in three ways. Firstly, this study established that the use of integrated interventions that incorporate the message, construal level, design elements and context-specific cues is the most effective way of addressing the multifaceted problem of food waste. This study therefore contributes to literature by confirming that the problem of food waste may not be addressed by only employing single message frames. The use of multiple food waste reduction message frames resonates with the principles of the integrated social marketing communication approach which advocates for social marketers to leverage on the synergistic effect of different messages to elicit behavioural change (Kim et al., 2019; Pearson and Perera, 2018).

**Figure 2.**
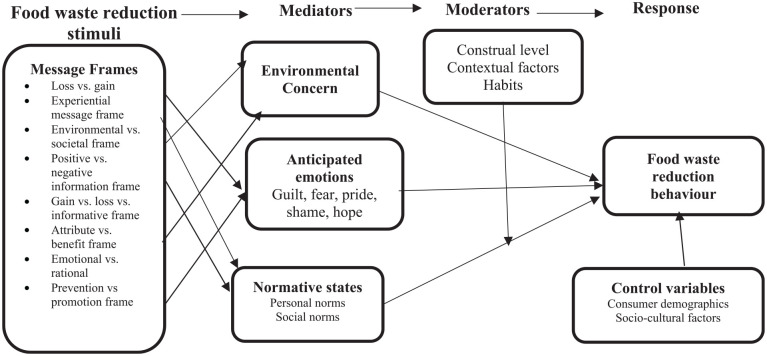
Proposed integrated model of food waste reduction. Source: Authors’ compilation.

Secondly, this study also contributes to literature by identifying novel factors that enhance the potency of food waste messages. Specifically, this study established that the effectiveness of food waste reduction messages depends on source credibility (Begho and Zhao, 2023), message design (Abos et al., 2024) and context manipulation (Antonschmidt and Lund-Durlacher, 2021; Dolnicar et al., 2020). Lastly, this study contributes to efforts aimed at addressing the inconsistent results reported in food waste reduction literature. We propose an integrated model that has the potential to account for more variance in food reduction behaviour. The proposed model includes food waste reduction stimuli, mediators, moderators and food waste reduction behaviour as the outcome variable. Figure 2 shows the proposed conceptual model.

### Social marketing implications

Messages that blame or shame consumers for wasting food were found to have a backfiring effect that triggers consumers to be reluctant to engage in food waste reduction behaviour (Birau and Faure, 2018). This result challenges policymakers and social marketers to shift from the use of negative to positive framed food waste reduction messages. This may be done by developing messages that positively complement the role of consumers in addressing food waste in a manner that counterbalance the goal of food waste reduction and customer satisfaction. To covey such messages, literature reviewed underscored the need to use credible sources in order to enhance message acceptance.

The effectiveness of food waste reduction messages was found to be dependent on how the message is designed and presented (Abos et al., 2024). Drawing from this result, social marketers should consider the use of visual graphical messages as they were found to be more effective in reducing food waste as compared to verbal messages. As recommended by Abos et al. (2024), participation of consumers in food waste reduction initiatives may be enhanced by presenting food waste messages in the form of experiential cues that enable consumers to take a participatory role.

The use of multiple food waste reduction message frames emerged as a growing trend from the literature reviewed. This result challenges social marketers to move away from the common practice of using single message frames as well as models developed from single theories. Social marketers may operationalise this finding by developing an integrated social marketing communication plan which incorporates multiple message frames ad interventions in order to develop compelling food waste reduction stimuli. The adoption of such an integrative approach has the potential to assist social marketers to convey and reinforce food waste reduction messages in a consistent, coordinated and cost-effective manner.

The prevalence of different construal levels associated with the perception of the problem of food waste was evident in literature reviewed. Against this background, segmentation, targeting and positioning should be considered as central to the development of social marketing intervention programmes. As suggested by Zhang et al. (2020), the degree of environmental concern and food waste mindfulness may be used as consumer profiling and segmentation variables. Some of the propositions that may be used to develop food waste reduction messages include debunking the prevalent myth in developing economies that environmental sustainability is not urgent and to reinforce the fact that food waste is a social issue and that it is morally wrong to waste food.

This study also revealed that entrenched habits are one of the major constraining factors of food waste reduction behaviour. Social marketers may respond to this challenge by developing intervention strategies that weaken the habitual behaviour of wasting food. To do this, they may rely on the insight from Ong et al. (2023) that underscore the need to harness the power of digital nudging through the use of social media or food sharing platforms. Drawing from Ong et al. (2023), social marketers may also seek to break the consumers’ food wasting habits by empowering them with food waste management skills including a focus on eco-innovations.

### Limitations and future research

There are three main limitations that are worth mentioning which point to the need for further research. Firstly, this study focused on messages that were employed to address food waste at consumer level. As food waste is characterised as a complex problem influenced by the interaction of many stakeholders in the food supply chain, future studies may focus on both downstream and upstream message frames in order to formulate holistic social marketing interventions. Secondly, this study only reviewed empirical studies and reports that were published between 2015 and 2024. Thus, there is a possibility that this study may have missed insights from other studies that were published outside the search period. Future studies may improve the findings of this study by having a more prolonged search period. Lastly, the majority of empirical studies reviewed used an experimental research design. Simulated models were mainly used and may be limited in terms of capturing critical context specific factors that influence food waste reduction behaviour. Future studies may address this shortcoming by using mixed methods incorporating real life research settings.

## Conclusion

An SLR was employed to understand the message frames employed to address the problem of food waste at consumer level. The use of multiple food waste reduction message frames was identified as a growing trend. This accentuates the importance of developing an integrated social marketing communication strategy that incorporates multiple message frames in order to convey food waste reduction messages in a consistent, coordinated and cost-effective manner. The prevalence of different construal levels associated with the problem of food waste was also evident in literature reviewed. Thus, market segmentation, targeting and positioning should be considered as central to the development of effective food waste reduction intervention programmes. This study revealed that entrenched habits are one of the major constraining factors of food waste reduction behaviour. Social marketers may respond to this challenge by developing intervention strategies that weaken the habitual behaviour of wasting food. This may be done by harnessing the power of digital nudging through the use of social media or food sharing platforms. Food waste was also characterised as a complex and multifaceted problem. In order to develop an integrated and coordinated strategy for addressing the problem of food waste, social marketers and policymakers may need to conduct a stakeholder mapping exercise. This has the potential to assist social marketers to identify key stakeholders with the requisite input to address the complex and multidimensional problem of food waste.
